# Analysis of global human gut metagenomes shows that metabolic resilience potential for short-chain fatty acid production is strongly influenced by lifestyle

**DOI:** 10.1038/s41598-021-81257-w

**Published:** 2021-01-18

**Authors:** David K. Jacobson, Tanvi P. Honap, Andrew T. Ozga, Nicolas Meda, Thérèse S. Kagoné, Hélène Carabin, Paul Spicer, Raul Y. Tito, Alexandra J. Obregon-Tito, Luis Marin Reyes, Luzmila Troncoso-Corzo, Emilio Guija-Poma, Krithivasan Sankaranarayanan, Cecil M. Lewis

**Affiliations:** 1 Laboratories of Molecular Anthropology and Microbiome Research, University of Oklahoma, 101 David L. Boren Blvd, Norman, OK 73019 USA; 2 Department of Anthropology, University of Oklahoma, Norman, OK 73019 USA; 3 Halmos College of Natural Sciences and Oceanography, Nova Southeastern University, Fort Lauderdale, FL 33314 USA; 4 Ministry of Health, Ouagadougou, Burkina Faso; 5 Centre MURAZ Research Institute, Bobo-Dioulasso, Burkina Faso; 6 Department of Biostatistics and Epidemiology, College of Public Health, University of Oklahoma Health Sciences Center, Oklahoma City, OK 73104 USA; 7 Département de Pathologie et Microbiologie, Faculté de médecine vétérinaire, Université de Montréal, Saint-Hyacinthe, QC J2S 2M2 Canada; 8 Département de médecine sociale et préventive, École de santé publique de l’université de Montréal, Montréal, QC H3N 1X9 Canada; 9 Centre de Recherche en Santé Publique (CReSP) de l’université de Montréal et du CIUSS du Centre Sud de Montréal, Montréal, QC H3N 1X9 Canada; 10 Center for Applied Social Research, University of Oklahoma, Norman, OK 73019 USA; 11 Centro Nacional de Salud Publica, Instituto Nacional de Salud, Lima, Perú; 12 Facultad de Medicina, Universidad Nacional Mayor de San Marcos, Lima, Perú; 13 Centro de Investigación de Bioquímica y Nutrición, Facultad de Medicina Humana, Universidad de San Martín de Porres, Lima, Perú; 14 Department of Microbiology and Plant Biology, University of Oklahoma, Norman, OK 73019 USA

**Keywords:** Microbial communities, Microbial ecology, Microbial genetics

## Abstract

High taxonomic diversity in non-industrial human gut microbiomes is often interpreted as beneficial; however, it is unclear if taxonomic diversity engenders ecological resilience (i.e. community stability and metabolic continuity). We estimate resilience through genus and species-level richness, phylogenetic diversity, and evenness in short-chain fatty acid (SCFA) production among a global gut metagenome panel of 12 populations (n = 451) representing industrial and non-industrial lifestyles, including novel metagenomic data from Burkina Faso (n = 90). We observe significantly higher genus-level resilience in non-industrial populations, while SCFA production in industrial populations is driven by a few phylogenetically closely related species (belonging to *Bacteroides* and *Clostridium*), meaning industrial microbiomes have low resilience potential. Additionally, database bias obfuscates resilience estimates, as we were 2–5 times more likely to identify SCFA-encoding species in industrial microbiomes compared to non-industrial. Overall, we find high phylogenetic diversity, richness, and evenness of bacteria encoding SCFAs in non-industrial gut microbiomes, signaling high potential for resilience in SCFA production, despite database biases that limit metagenomic analysis of non-industrial populations.

## Introduction

Lifestyle alterations have repeatedly coincided with biological changes throughout the human past^1^ and this is particularly true for how industrialization changed the relationship between humans and our resident microbes^2^. Compared to industrial human gut microbiomes, non-industrial gut microbiomes have higher genus and species richness, functional enrichment of amino acid metabolism, greater diversity of genes involved in complex carbohydrate metabolism, and higher amounts of short chain fatty acids (SCFAs) in stool^3,4^. These trends have been linked to plant-based diets rich in fibers, infrequent consumption of highly processed foods, and low exposure to pharmaceutical drugs, such as antibiotics, in non-industrial populations^5^.

Higher diversity in the gut microbiome is typically considered healthy, all other factors being equal^2^, which would imply that a non-industrial gut is healthier than the industrial gut, in the absence of confounding variables, such as bacterial pathogens^6^, eukaryotic parasites^7^, and malnutrition^8^. Yet, commonly used diversity statistics oversimplify more complex microbial associations. Ecological approaches that provide context for microbe-microbe interactions, and present insights into how taxonomic shifts influence microbial and host metabolic processes, are making progress towards mitigating this issue. Taxa-gene relationships are at the heart of deeper ecological understandings of human microbiomes and can be assessed through functional diversity and redundancy^9,10^. Functional diversity, which is similar to the macroecological concept of response diversity^11^, refers to the abundance and phylogenetic diversity (PD) of taxa that encode specific genes. It conceptualizes structure–function relationships in the microbiome by tying together taxonomic and metagenomic gene abundance data. Similarly, redundancy can be thought of as the total number of taxa encoding a function, as well as how evenly the production of any given protein is spread amongst taxa.

Functional diversity can be multi-layered, ranging from a fine-tuned focus on individual genes to a broad genome-wide approach. Gene-centric approaches present the opportunity for niche-specific interpretations, while a broader approach allows for study of how entire microbiomes may shift in the face of outside perturbations. No matter the depth and focus of study, high functional diversity is found in microbiomes where phylogenetically diverse bacteria encode the same functions. Under an idealized model, phylogenetically diverse taxa will have an equal contribution to gene production, leading to high redundancy. Functional diversity and redundancy are intertwined and together estimate microbiome resilience. Shifts in taxonomic abundance are less likely to alter the functional potential of a resilient community because any given function is encoded by a wide range of bacteria and production is distributed between these diverse taxa. Consequently, the loss of one phylogenetic branch of bacteria within the ecosystem will not cause a loss of function that those bacteria encode; however, communities with low functional diversity and redundancy may suffer ecosystem-wide functional changes during minor taxonomic perturbations. Accurately quantifying functional diversity is therefore a necessary part of ecologically minded microbiome research because it more deeply describes how structure–function relationships influence resilience in a microbiome.

SCFA synthesis is the most intuitive area of study for understanding ecological differences between industrial and non-industrial gut microbiomes, given the trends attributed to high-fiber diets among non-industrial populations. SCFAs are important byproducts of microbial metabolism and dietary fiber fermentation in the human gut. The three most prominent SCFAs in the human gut (acetate, butyrate, and propionate) are vital for maintenance of tight junction integrity between epithelial cells in the gastrointestinal (GI) tract, serve as an energy source for colonocytes, and signal immune cells, amongst a number of other functions^12,13^. Unsurprisingly, variation in SCFA abundance is a classic link to human health. For example, high butyrate levels are found to decrease diastolic blood pressure via regulating inflammation, and acetate abundance is tied to appetite, thus impacting metabolic regulation^12,13^.

**Figure 1:**
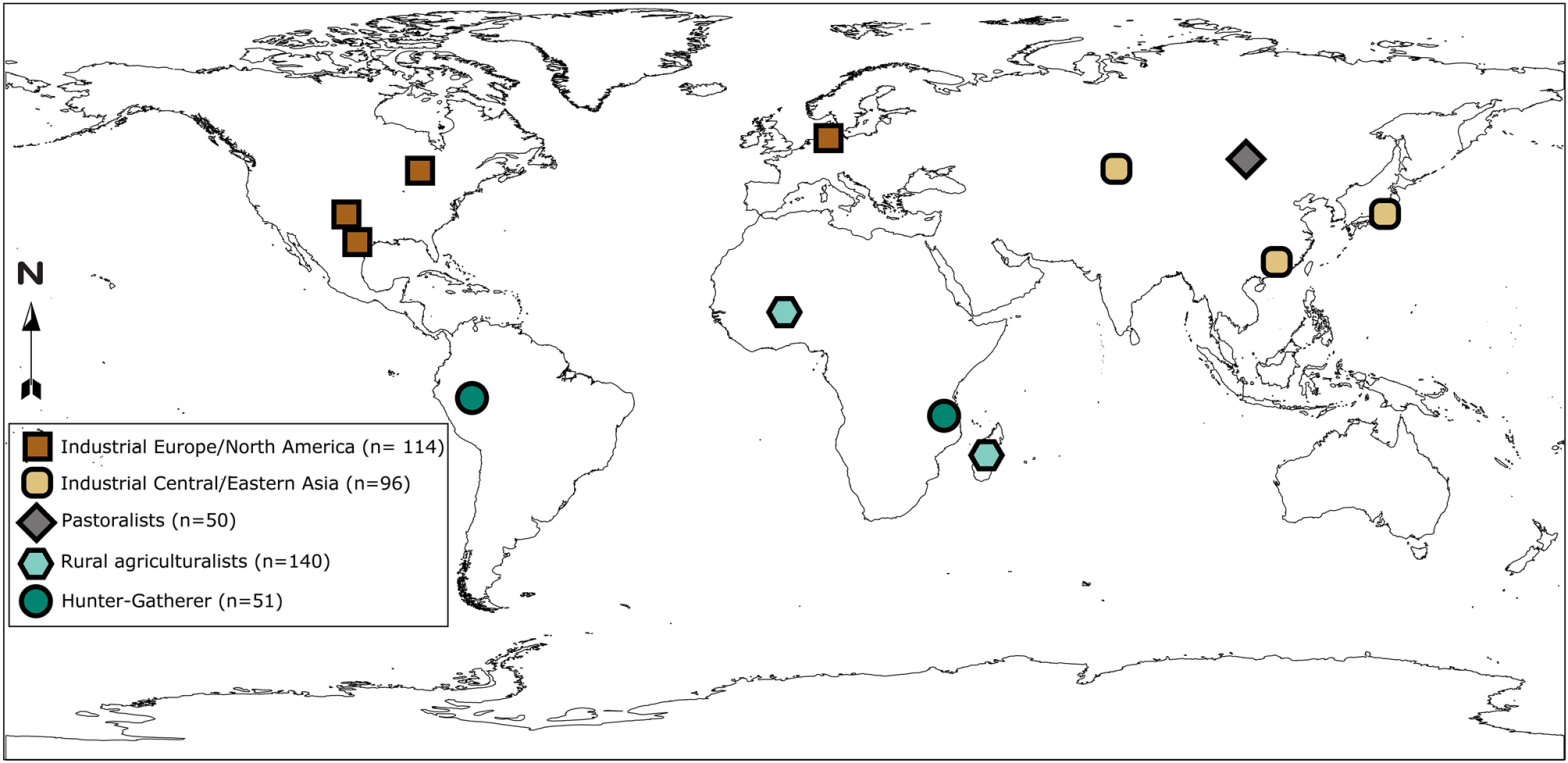
Map of human gut microbiome metagenomes analyzed. Analyzed microbial metagenomes originated from the following populations/datasets: Industrial North American/European—Human Microbiome Project^25^ (Missouri, Texas—USA, n = 50), Oklahoma^3^ (USA, n = 21), Northern Europe^27^(n = 43) ; Industrial Central/East Asia—China^21^ (Guandong Province, China, n = 38), Tokyo^20^ (Japan, n = 32), Astana^24^ (Kazakhstan, n = 26); Pastoral—Mongolia^23^ (Khentii Province, n = 50); Rural Agriculturalist—Burkina Faso (n = 90), Madagascar^26^ (n = 50); Hunter Gatherer—Matses^3^ (Peru, n = 25), Hadza^22^ (Tanzania, n = 26).

Studying SCFA functional diversity is particularly intriguing as it provides a line of evidence as to whether estimates of taxa/gene diversity and ecological resilience are concordant. Research suggests that non-industrial populations have high SCFA abundance, which is attributed to dietary composition^4,5^. It is assumed that non-industrial gut microbiomes bear an ecology that is resilient for SCFA production due to high overall taxonomic diversity and high SCFA levels in stool, but this has not been demonstrated. We address this gap by analyzing functional diversity and redundancy of SCFA synthesis genes in metagenomic datasets from 12 different populations (Fig. 1). Datasets were grouped together based on similarity of lifestyle because diet and lifestyle have been shown to be major drivers of microbiome diversity^2,14^, resulting in the following general lifestyle groups: industrial (European/North American and Central/East Asian), pastoral, rural agricultural, and hunter-gatherer populations. Industrial populations were split between North America/Europe and Central/East because previous work has suggested variation in gut microbiome composition between these regions^15^, although more work needs to be done in this area. We chose to evaluate SCFA genes that are involved in end-stage synthesis in different pathways for each SCFA: acetate kinase (*ackA*) for acetate^16^, butyrate kinase (*buk*) and butyryl-CoA: acetate CoA transferase (*but*) for butyrate^17,18^, and methylmalonyl-CoA decarboxylase *(mmdA)*, lactoyl-CoA dehydratase (*lcdA*), and CoA-dependent propionaldehyde dehydrogenase (*pduP*) for propionate^17,19^. These genes are known to be encoded by a diverse range of bacteria (Table S1), which permits ecological investigation into the resilience of SCFA production. Our study includes previously published metagenomic datasets from industrial and non-industrial populations^3,20–27^, as well as novel gut microbiome metagenomic data generated from fecal samples collected from rural agriculturalists living in central Burkina Faso (n = 90). We functionally profiled these metagenomes using HUMAnN2^28^.

Each gene we analyzed is involved in terminal or near-terminal steps of production of each respective SCFA^16–19^. For analytical purposes, taxonomic-gene abundance for *but* and *buk* were combined for butyrate and data for *mmdA, lcdA,* and *pduP* were aggregated for propionate. Response diversity was estimated through genus and species richness and phylogenetic diversity, while the Gini-Simpson index and Hill Numbers were used to assess redundancy by documenting evenness in the community. For our purposes, the Gini-Simpson index^29^ represents the probability that two sequencing reads originate from different taxa, and therefore values close to 1 represent a community with a high diversity of taxa encoding the SCFA, while values close to 0 indicate that the SCFA is encoded almost entirely by one taxon. Hill numbers^30^ are a diversity measure that allows for interpretation of effective taxonomic richness at different levels: at diversity order 0 the Hill number value is equal to the total number of taxa (i.e. richness), at diversity order 1 the Hill number value is the effective number of commonly occurring taxa, and at diversity order 2 the Hill number value is the effective number of dominant taxa. A higher number of common and dominant taxa indicates resilience, as a community with minimal common and dominant taxa would be prone to function elimination if those few common/dominant taxa are removed from the community. Therefore, Gini-Simpson and Hill number investigations permit analyzing how evenly SCFA production is distributed between taxa and present opportunities for resilience estimation.

## Results

**Figure 2:**
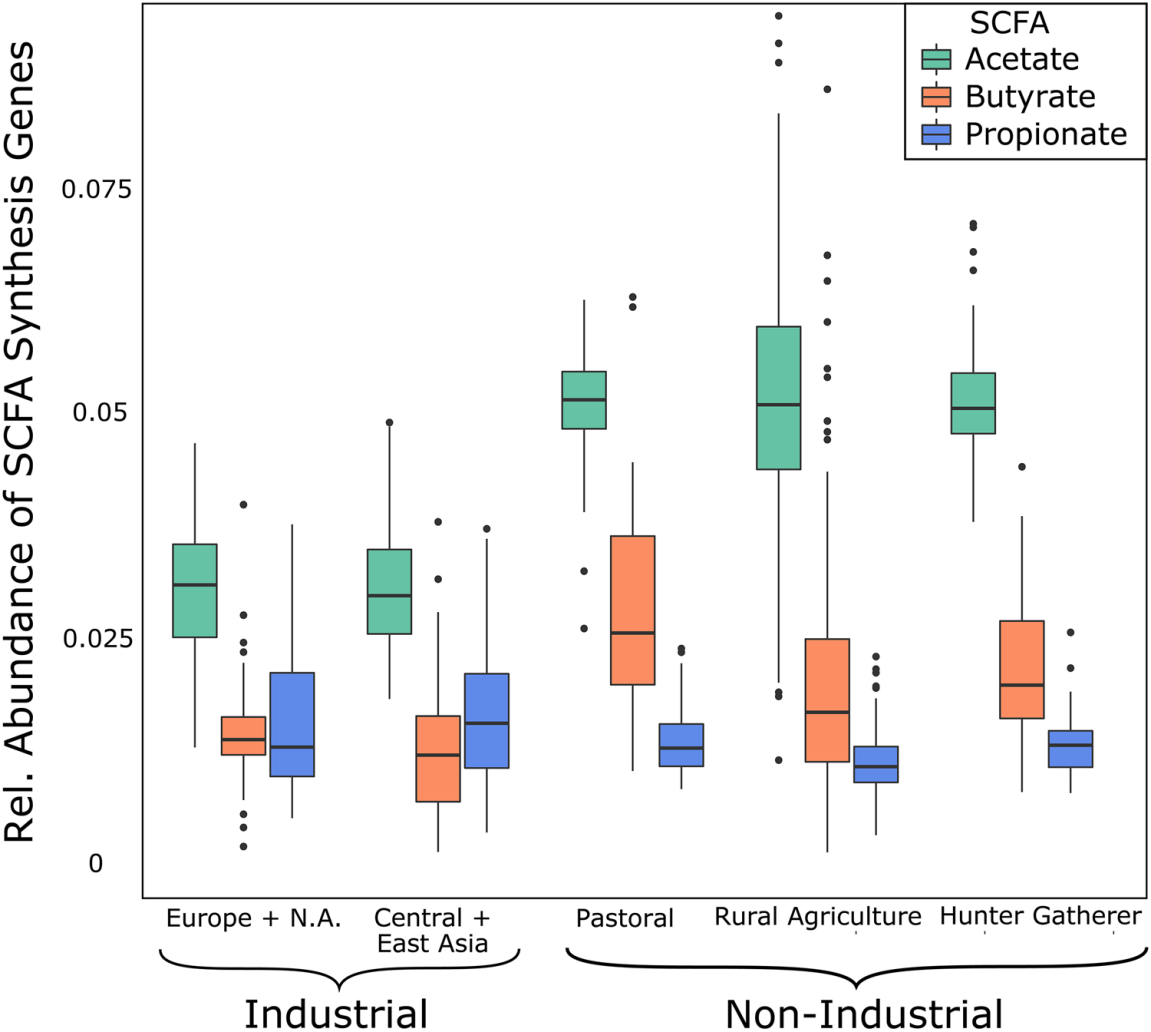
Relative abundance of SCFA genes compared between lifestyle categories. Acetate and butyrate are significantly higher in each of the non-industrial populations as compared to the industrial populations. Propionate is significantly lower in the rural agriculturalists compared to all other lifestyles, whereas butyrate was significantly lower in abundance in rural agriculturalists compared to hunter gatherers and pastoralists. There were no significant differences between the European/North American (Europe + N.A.) and Central/East Asian industrial population, and likewise, no significant differences between pastoralists and hunter-gatherers for any of the SCFA genes. FDR adjusted p-values for all statistical comparisons can be found in Data File S1.

Independent of lifestyle, acetate synthesis was significantly more abundant than the other two SCFAs (p-value < 8 × 10^–84^) and butyrate was more abundant than propionate (p-value < 6 × 10^–8^, Table S2). The overall higher abundance of butyrate compared to propionate across the full dataset is driven by the non-industrial populations, as propionate and butyrate are at similar abundance in industrial populations (Table S2). The relative abundance ratio of SCFA synthesis genes of acetate:butyrate:propionate (mean = 0.600 [standard error = 0.001] : 0.215 [SE = 0.001]: 0.184 [SE = 0.001]) (Table S2), supports the previous finding of a 60:20:20 ratio of SCFA molarity in stool^12^; however, non-industrial populations have been reported to have higher a concentration of propionate than butyrate^31,32^. Comparing between lifestyles, acetate (FDR-adjusted p-value < 3 × 10^–18^, n = 451) and butyrate (FDR-adjusted p-value < 0.004, n = 451) synthesis genes were more abundant in each of the non-industrial populations (Fig. 2). Propionate synthesis genes were similar between lifestyle groups, with the exception of being at significantly lower abundance in rural agriculturalists compared to all lifestyles (FDR-adjusted p-value < 0.02, n = 451, Fig. 2). Within the general lifestyle categories, the rural agriculturalists had significantly lower abundance of butyrate and propionate compared to hunter-gatherers and pastoralists (FDR-adjusted p-value < 0.02, n = 241) while there was no significant difference between European/North American industrial and Central/East Asian industrial populations for any of the SCFA gene groups (FDR-adjusted p-value > 0.05, n = 210) (Fig. 2).

**Figure 3:**
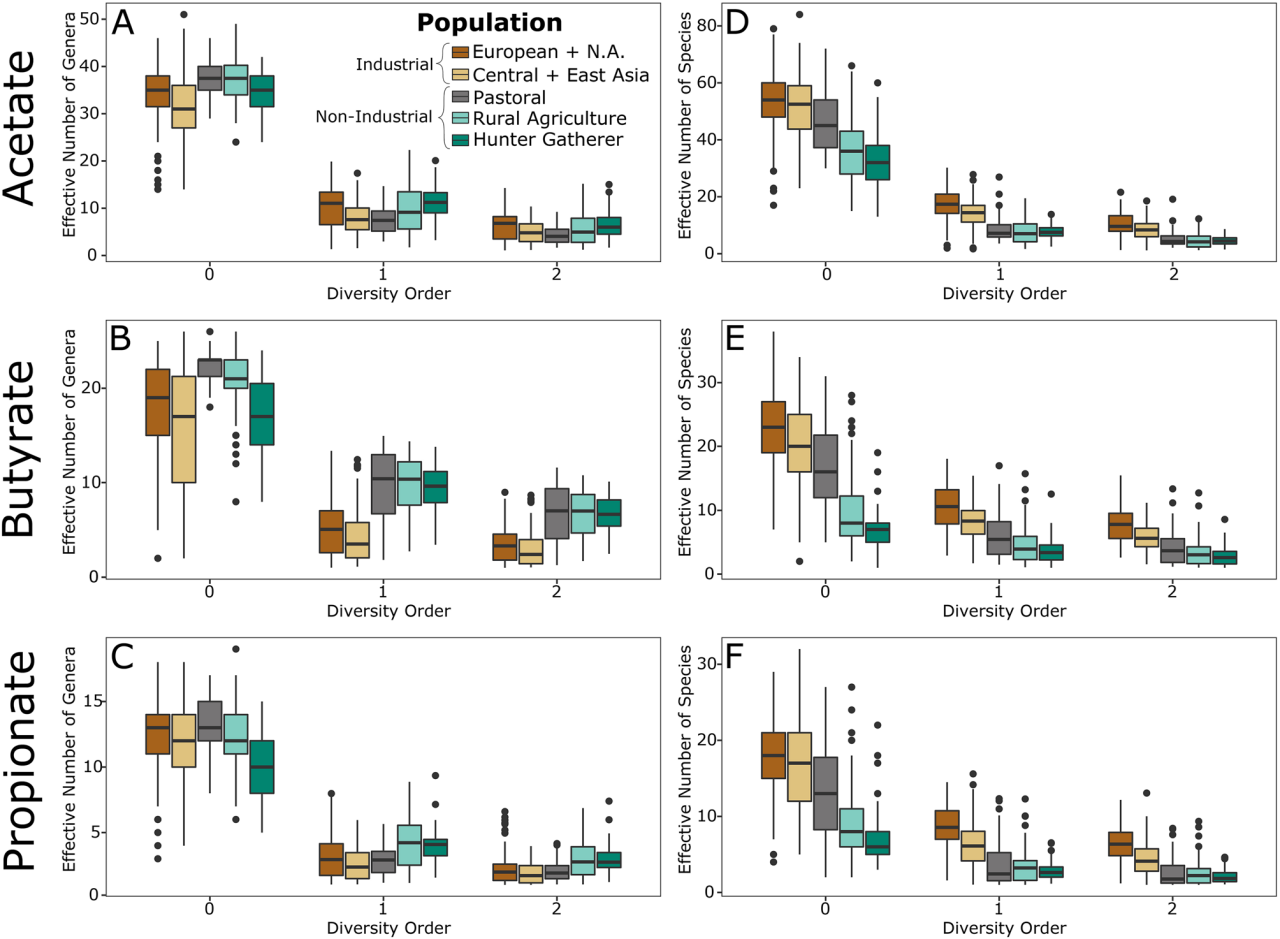
Hill Numbers for SCFA-encoding Taxa. Effective number of genera (**A**–**C**) and species (**D**–**F**) for each SCFA as determined through Hill numbers at diversity order 0 (richness), 1 (number of common taxa), 2 (number of dominant taxa). Non-industrial populations have significantly greater genus richness for acetate and butyrate but not propionate, while species richness is significantly lower in non-industrial for all SCFAs. Rural agriculturalists and hunter-gatherers have significantly higher number of common and dominant genera than both industrial populations for butyrate and propionate, but only greater diversity than the Central + East Asian dataset for acetate at diversity order 1. This means the distribution of SCFA production in non-industrial populations is more even. The number of effective number of species is significantly lower in the non-industrial populations for each SCFA. FDR adjusted p-values for all statistical comparisons can be found in Data File S1.

The pastoralist and rural agricultural populations have significantly higher genus richness (Hill diversity order 0) for acetate and butyrate synthesis compared to the industrial populations (FDR-adjusted p-value < 0.006, Fig. 3A,B, n = 401); however, hunter-gathers only have significantly greater abundance than the Central/Eastern Asia population for genera involved in acetate synthesis (FDR-adjusted p-value = 0.014, Fig. 3A, n = 261 ). Hunter-gatherers have significantly lower genus richness for propionate synthesis compared to both the industrial and non-industrial populations (FDR-adjusted p-value < 3 × 10^–5^, Fig. 3C, n = 261). The high genus-level richness observed in the non-industrial populations for acetate and butyrate is not observed at the species level, as every non-industrial population has significantly lower species richness for each SCFA gene (FDR-adjusted p-value < 0.05, Fig. 3D–F, n = 451). Additionally, the rural agricultural and hunter-gatherer populations have significantly lower species richness than the pastoralists for acetate, butyrate, and propionate (FDR-adjusted p-value < 3 × 10^–4^, Fig. 3D–F, n = 241). Similar to species richness, we observed lower species phylogenetic diversity (PD) in the non-industrial populations, but the effect sizes were not as large as in species richness (Fig. S1A–C). The hunter-gatherers had significantly lower PD for each SCFA, compared to the industrial populations (FDR-adjusted p-value < 0.04, Fig. S1C, n = 261); however, the rural-agriculturalists only have lower PD for butyrate and propionate (FDR-adjusted p-value < 5 × 10^–8^, Fig. S1C, n = 350) and there were no significant differences between pastoralists and industrial populations for species PD. The small drop-off in species PD, compared to species richness, suggests there are a large number of closely phylogenetically related species in the industrial gut microbiome. We found *Bacteroides* and *Clostridium,* which are found at high abundance in industrial gut microbiomes, to have up to nine species encoding SCFAs, while known SCFA producers at high abundance in non-industrial gut microbiomes (*Prevotella*, *Faecalibacterium*, and *Phascolarctobacterium)* only had one or two species per each genus (Table S3).

Genus evenness, as gauged through effective number of genera at Hill diversity order 1 (number of common genera) and diversity order 2 (number of dominant genera), tell a unique story for each SCFA. Hunter-gatherers and rural agriculturalists have higher effective number of common and dominant genera compared to the industrial populations for butyrate and propionate, but diversity is only greater in non-industrial populations at Hill number 1 for acetate when compared to the Central/East Asian population (FDR-adjusted p-value < 0.02, Fig. 3A–C, n = 451). Additionally, for Hill numbers 1 and 2 the pastoralists have significantly higher diversity than the industrial populations for butyrate (FDR-adjusted p-value < 3 × 10^–10^, n = 260) but lower diversity than the European/North American industrial population for acetate (FDR-adjusted p-value < 0.003, n = 260). These results demonstrate the industrial populations have only a few common and dominant genera encoding SCFAs, indicating that they are prone to loss in SCFA production if those common/dominant genera are lost. At the species level, evenness is significantly lower for each SCFA in every non-industrial population compared to the industrial groups (FDR-adjusted p-value < 0.05, Fig. 3D–F, n = 451). Similar to the richness and Hill numbers, the Gini-Simpson index is higher in non-industrial populations at the genus-level but lower at the species level (Fig. S2).

The discrepancy between genus and species results, particularly the drastic drop-off in diversity in non-industrial populations at the species level suggests a loss of information during annotation of non-industrial gut metagenomes. To probe this further, we assessed the proportion of total DNA fragments that mapped to a gene between lifestyles, as well as the proportion of those gene-mapped fragments that were classified to a taxon. After normalizing gene abundance to genes per 1 million DNA molecules, genes are positively identified from approximately 75% of DNA fragments in the industrial populations, but gene identification drops to about 65% of DNA fragments in non-industrial populations (p-value < 5.41 × 10^–15^, n = 451). The stratified HUMAnN2 output provides abundance of genes matched to bacteria at different taxonomic levels (‘classified’), as well as gene abundance not accounted for by any taxon (‘unclassified’). Across all genes identified in each metagenome, there is a significant decrease in the proportion of gene abundance that is classified to a bacterium at each taxonomic level from industrial to non-industrial populations (FDR-adjusted p-values: phylum < 5.13 × 10^–9^, family < 6.27 × 10^–9^, genus < 4.24 × 10^–8^, species < 8.25 × 10^–8^; Fig. S3, n = 451). Only 25–30% of gene abundance is classified to a species in hunter-gatherers and rural agriculturalists but upwards of 65% to 75% of genes are classified to species in industrial populations. Therefore, there are significantly fewer genes identified in non-industrial metagenomes and this loss of information is compounded by substantially worse identification of the taxa that encode those genes in non-industrial gut metagenomes.

**Figure 4:**
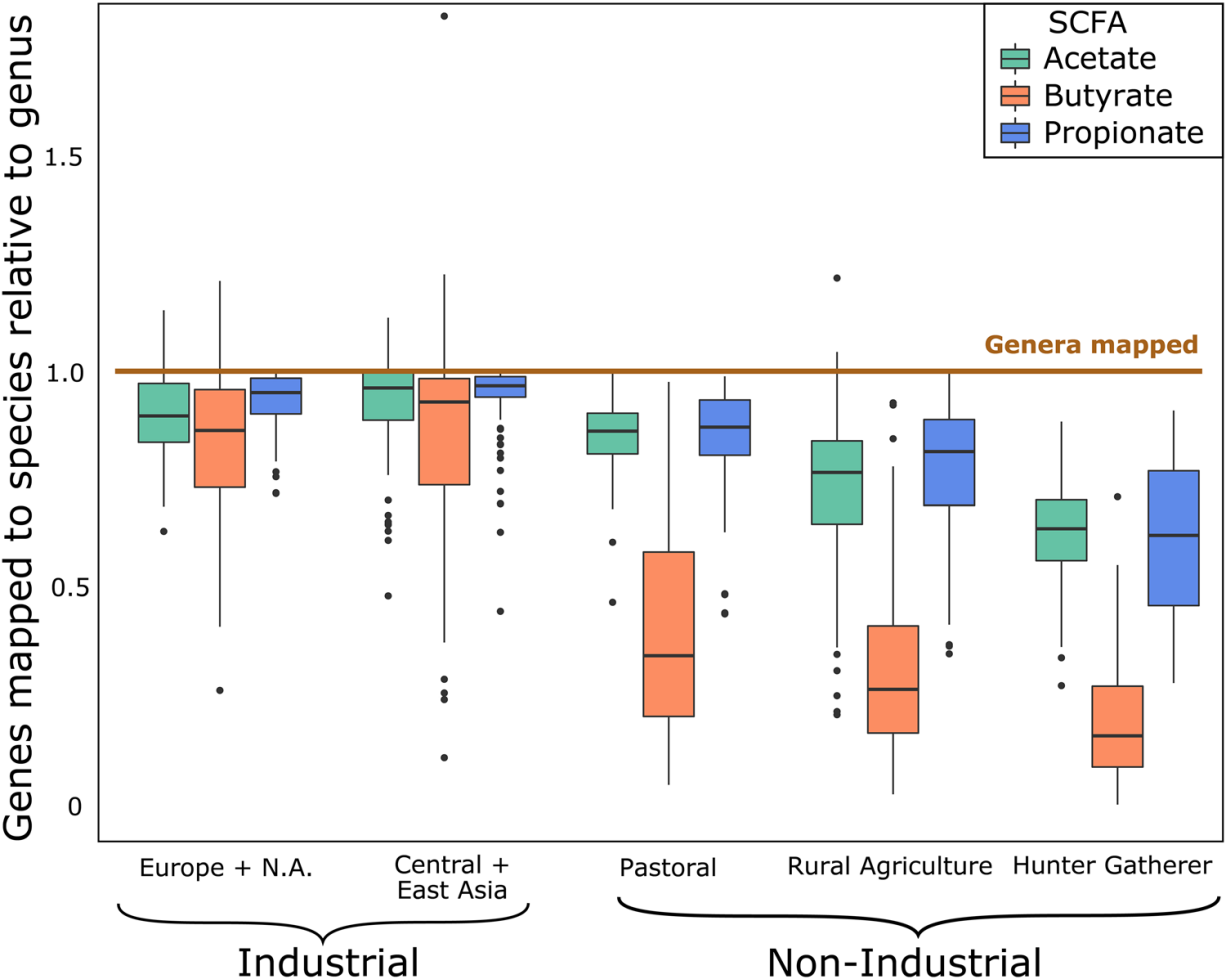
Genus: species relative mapping index. Relative index of genes mapped to a taxon at the species level (each box) normalized to genes mapped to a taxon at the genus level (1.0). In the non-industrial populations, there is a significant drop-off in the genes mapped to a taxon at the species level for each gene (FDR-adjusted p-value < 0.001, n = 241), while in industrial populations the rate of mapping is similar at the genus and species level (FDR-adjusted p-value > 0.05, n = 210). Values above 1.0 are due to genes that map to taxa at the species level but not the genus level. This is the result of candidate species (primarily in Lachnospiraceae family) that are annotated at the species level but have a missing annotation at the genus level. This is more common in the industrial populations in our dataset, once again likely due to bias that favors for industrial datasets. FDR adjusted p-values for all statistical comparisons can be found in Data File S1.

The afore-mentioned pattern is replicated for each of the SCFA synthesis gene groups, as there is significantly lower classification percentage at every taxonomic level in the non-industrial populations (FDR-adjusted p-value < 0.05, Fig. S4A–C, n = 451), with the exception of the pastoral populations for all taxonomic levels for acetate and at the phylum level for butyrate. Nevertheless, there are interesting trends for each of the SCFAs. Even though acetate is the most abundant SCFA, the classification percentage is essentially the same for the other two SCFAs (Fig. S4). For butyrate, the species-level information for non-industrial populations is by far the lowest of any of the SCFAs (Fig. S4B). All taxonomic levels have poor classification in rural agriculturalists and hunter-gatherers for propionate; phylum-level classification in hunter-gatherers is nearly half of species-level classification in industrial populations (Fig. S4C). A closer examination of genus and species-level mapping for each SCFA reveals a steep drop-off from genus-level classification to species-level classification in the non-industrial populations compared to industrial populations. In industrial populations the proportion of genes mapped to genera and proportion of genes mapped to species is similar (FDR-adjusted p-value > 0.05; Fig. 4, n = 210), while in rural agriculturalists and hunter-gatherers only 25–75% of genes mapped to genera are also mapped to species. The drop-off in mapping at the species level is significantly worse in the non-industrial populations for each SCFA (FDR-adjusted p-value < 0.02; Fig. 4, n = 451).

## Discussion

Our results are consistent with a non-industrial gut harboring a more resilient ecology with respect to SCFA production, while the industrial gut ecology would be vulnerable to disruption of such pathways, yet the pattern is complex and nuanced. The increased gene abundance in non-industrial populations and overall ratio of acetate:butyrate:propionate generally agrees with previous studies of SCFAs^5,12^. Similarly, the higher genus-level diversity of bacteria encoding acetate, compared to the other SCFAs, is expected and matches studies that have documented the taxa that encode different SCFAs^13,17,19^. The overall high richness, high diversity at Hill numbers 1 and 2, and high Gini-Simpson indices found in non-industrial populations at the genus level indicates a highly diverse and evenly distributed production of SCFAs. From an ecological perspective, uneven production of SCFA dominated by a few bacteria in industrial gut microbiomes means lower functional diversity and less redundancy, which ultimately leads to an expectation of decreased resilience. In other words, this study finds that industrial gut microbiomes are at a higher risk of reduced SCFA production because SCFA synthesis is dominated by only a few genera. Given the lower resilience, factors that disrupt the gut ecology are expected to have a more extreme consequence to those living an industrial lifestyle.

While there is an overall trend of increased genus-level functional diversity and redundancy for SCFA production in non-industrial populations, variation exists when examining the SCFAs and populations individually. At the genus-level, the pastoral and rural agricultural populations have increased richness of genera encoding genes involved in acetate and butyrate synthesis, while there is similarity across the different lifestyles for genus richness for propionate encoding taxa. Although hunter-gatherers have similar, or lower, genus richness as industrial populations, they have significantly higher diversity at Hill number orders 1 and 2 and Gini-Simpson indices for butyrate and propionate. Additionally, the pastoralists have a generally similar profile to the industrial populations for acetate and propionate Hill number diversity, as well as similarity to the industrial populations in species PD, which may be linked to this pastoralist group having a diet similar to some industrial populations; namely, a diet high in dairy and red meat consumption, coupled with few dietary sources of plant-derived fibers^23^. This paints a complex picture. Non-industrial populations have a high diversity of genera encoding butyrate synthesis, and butyrate production is spread more evenly across genera in non-industrial populations than in industrial populations. Hunter-gatherers and rural agriculturalists have significantly greater evenness of propionate production, even though they have fewer number of total genera encoding this SCFA. Finally, the richness and evenness of genera encoding acetate is similar between industrial and non-industrial populations. Ecologically, we would expect the industrial populations to be less resilient for production of butyrate and propionate when faced with a shift in taxonomic composition, while non-industrial populations may be only marginally more resilient for acetate production compared to industrial populations. Intriguingly, SCFA relative abundance does not appear to correlate to resilience profile. Acetate and butyrate are significantly more abundant in non-industrial populations but only butyrate shows much stronger resilience profile for non-industrial populations. Additionally, propionate is slightly more abundant in industrial populations, although not significantly, yet our results indicate greater resilience in non-industrial groups for propionate production. This indicates that measuring only total gene, and/or molar, abundance is not enough to make statements about metabolic processes in the human microbiome; rather, ecological approaches are necessary to understand diversity in functional potential of the human microbiome.

The increased species-level alpha diversity in industrial populations initially runs counter to the genus-level results but the genus and species level results ultimately yield similar interpretations after accounting for ecology and ascertainment bias, as discussed below. The substantially higher species richness in industrial populations is striking; however, the differences in PD between industrial and non-industrial populations are not nearly as extreme. This means that the high species richness in the industrial populations is driven by species that are closely phylogenetically related. Indeed, we observed SCFA producing genera found at high abundance in industrial populations (*Bacteroides* and *Clostridium*) to have up to nine species encoding SCFAs, while highly abundant non-industrial genera only have one or two species. Therefore, what first appears to indicate high species-level ecological resilience in SCFA production in the industrial populations is actually the result of closely related species performing the same function. It follows that closely related species may be prone to changes in abundance or even elimination after certain types of ecosystem shift events. For example, narrow-spectrum antibiotics^33^ and exposure to various xenobiotic compounds that lead to variable bacterial metabolic responses^34^ are events that can affect a limited range of bacteria and lead to shifts in microbial abundance and metabolic activity. While this result has ecological implications, it is also likely the result of historical trends of microbiology research. Bacterial taxa at high abundance in non-industrial gut microbiomes have not been a focus of microbiological isolation and species identification until recently; therefore, we expect more species to be identified from non-industrial gut microbiomes in the future^35^. Additionally, classification of bacteria into distinct genera and species is undergoing a revolution in the genomic era^36^ meaning that the high number of species classified to *Bacteroides* and *Clostridium* may ultimately be reclassified to different genera. Nevertheless, the fact that we observe a large jump in species richness, but only a minor increase in species PD, in the industrial gut microbiomes suggests that the high industrial species richness is driven by closely related species and therefore, results in the same interpretation as the genus richness results: diversity is high in non-industrial populations.

Ascertainment bias extends to the databases used to identify taxa and genes: fewer genes were identified in non-industrial populations and a smaller proportion of these genes can be linked back to bacteria at every taxonomic level, in non-industrial gut microbiomes. In some cases, such as butyrate synthesis genes, less than 10% of genes are identified to species for non-industrial populations, while over 50% of such identifications were possible for industrial populations. A decreased ability to identify the genus and species encoding SCFA synthesis genes in non-industrial populations means that the ecological metrics underestimate the true ecological diversity of these genes. Moreover, the drop-off in classification from the genus to the species level was significantly greater in non-industrial populations compared to industrial populations. This drop-off means a much lesser ability to identify species compared to genera in non-industrial populations, which helps explain why species diversity was substantially lower in non-industrial populations. Nevertheless, the statistically significant differences observed at the genus-level send a strong signal of the high functional diversity, and potential resilience, of SCFA synthesis genes in non-industrial gut microbiomes.

The metagenome-wide poor performance in terms of gene identification and classifying SCFA genes to genera and species indicates a bias in reference databases that underrepresents diversity in non-industrial gut microbiomes, which is unsurprising. Bias is expected because the vast majority of human gut microbiome studies have used samples from industrial populations. There is an immense challenge in including non-industrial communities in biomedical research, including recruiting research participants, sustaining longitudinal sampling, building culturally appropriate community relationships, and even securing transport of samples^35^. This has resulted in comparatively few metagenomic studies of human gut microbiomes from non-industrial settings^35^. Nevertheless, our data demonstrate the extent of this bias and how it can hinder more in-depth study of human gut microbiome health. Given this sizable ascertainment bias favored industrial populations, the non-industrial populations are likely even more diverse, more resilient, than our databases can sufficiently characterize, making our genus-level results even stronger. Without a serious investment to include such populations, the characterization of microbiomes will remain naive to the ecological breadth of the core, healthy, human gut. Imagine studying forest ecology, with only city parks at your disposal. This has been, overwhelmingly, the analogous practice of human microbiome research.

The relative lack of microbiome studies with non-industrial populations means an underrepresentation of not only metagenomic data and genome annotation but also fewer opportunities for cultivation and validation of novel species of bacteria. This ultimately leads to an inequality in the depth to which researchers can describe microbiome samples from non-industrial communities, compared to industrial microbiomes, as diverse groups of novel taxa may be grouped into a single group of “unknown” or “unclassified” bacteria^35^. Similarly, an incomplete picture of microbial functional potential means that genes may be misidentified or even unannotated completely. Unknown taxa and misidentified genes may be playing key roles in ecological and metabolic processes but researchers are unable to confidently identify them, let alone make statements about their importance in a microbial ecology^35^. Recent human gut microbiome metagenome studies from diverse populations will undoubtedly improve database representation but the number of studies and metagenomic samples from non-industrial populations still pales in comparison to industrial gut microbiomes^26,35,37,38^.

Limitations in annotating the full extent of microbial diversity impacts health research. Recently proposed ‘Microbiota Insufficiency Syndrome (MIS)’^2^ postulates that, while the microbiome has adapted to industrialization, these adaptations are maladaptive to human health. The decreased phylogenetic diversity and loss of specific taxa (e.g. Prevotellaceae, Succinivibrionaceae, and Spirochaetaceae) observed in industrial gut microbiomes may contribute to the increase in non-communicable chronic diseases found at higher prevalence in industrial populations. The root cause of MIS in industrial populations is undoubtedly multifactorial; however, diet is suggested to play a major role^2^. This syndrome is compelling and we postulate that this insufficiency precisely rests on the stability of functional capacity. Our findings of decreased resilience in industrial populations, as well as species-level diversity driven by a few closely related species, fits in well with MIS. Low resilience in SCFA production may ultimately manifest itself as altered colonocyte function and/or autoimmune disruptions (both symptoms of MIS) due to a decrease in SCFA bioavailability after a group SCFA-producing bacteria were wiped-out during an ecological shift, such as antibiotic or xenobiotic exposure. Similar to MIS, diet is likely to play an important role in SCFA resilience. The non-industrial populations studied in this paper consume much more fiber than industrial populations, on average^3,5,14,25,26^, and microbial fermentation of dietary fibers is a major source of SCFAs in the human digestive tract^39^. A diet poor in dietary fiber means less substrate for microbial fermentation and therefore less SCFA production and also higher competition for that fiber, potentially resulting in competitive exclusion and less microbial diversity. Nevertheless, if we are unable to fully characterize and annotate non-industrial gut microbiomes then we will be unable to paint a complete picture of MIS. Currently, we have confidence that there is a wealth of undiscovered resilience in non-industrial gut microbiomes. Once we describe the extent of this diversity/resilience, through increased sampling and focus on partnerships with research institutes in industrializing countries, we will have a more complete picture of MIS and possibly develop therapeutic approaches to combat non-communicable chronic diseases related to the human gut microbiome.

Improved sampling, metabolic profiling, and annotation will not only improve our understanding of SCFA resilience, but it will also permit more detailed picture microbiome wide resilience. Our work shows the value of focusing on specific SCFA genes, due to their importance in human biology and previously reported variation in SCFA molar abundance between industrial and non-industrial populations^31,32^; however, future work will undoubtedly add to our findings. One avenue for future work is through analyzing SCFA molar concentrations in fecal samples in a longitudinal setting and comparing these results to predicted SCFA resilience from metagenome panels. Unlike genomic data, where we can infer about SCFA production potential via taxonomic diversity, one-time measures of fecal SCFA molar concentrations will not inform about future resilience because SCFA molar concentrations carry no information about which taxa produce each SCFA. Longitudinal SCFA concentration and metagenomic data from non-industrial populations, or animal models, is necessary to inform about SCFA resilience and production in diverse lifestyles. Another avenue for future work is to focus resilience analysis on other microbiome functions of interest, such as resilience of antibiotic resistance genes and amino acid biosynthetic pathways. These valuable studies would be valuable for comparing microbiome resilience dynamics for different functions, with the caveat that there is sufficient genomic annotation data to yield interpretable results.

Lack of sample diversity is not unique to human microbiome research, as human genetics research has been grappling with this very issue for decades. In 2009, 96% of individuals included in human genome-wide association studies (GWAS) claimed European ancestry, as compared to 78% in 2019^40^. Thus, while there have been improvements, GWAS clearly fail to reflect the breadth of human diversity. Incorporating diverse populations in human genome and microbiome research has the potential to greatly benefit the scientific community’s understanding of human biology and develop treatments that are based on human diversity rather than European-ancestry genetics and microbiomes. A key component of increasing representation in genetics and microbiome studies is that these studies are designed as partnerships with minority and/or indigenous communities in a manner that builds both trust between the community and researchers, as well as facilitates the ability for the sample donors to exercise their rights on how data are treated and shared^41^.

## Materials and methods

### Study design

#### Sample size

Datasets were chosen because they represent a wide diversity of lifestyles, have a minimum of 20 samples per population, and were sequenced to an average read depth of 10 million reads per sample. We used 20 samples as a threshold based on previous research^42^ showing that at least 20 samples per population are required for the types of ecological analyses pursued in this study. Similarly, 10 million reads was chosen as a threshold to allow for sufficient read depth to attain coverage of as many genes as possible from each metagenome. The number of reads generated for the Burkina Faso samples is available in Data File S2 and the SRA accession number and number of reads analyzed for the comparative datasets are provided in Data File S3.

#### Data inclusion/exclusion criteria

For datasets with available metadata, we included only healthy adults (i.e. non-obese BMI, non-diabetic) in the analysis; however, children were included in the Matses and Hadza datasets due to limitation in sample size.

#### Outliers

Outliers were included in all analysis.

#### Research objectives

Our research objective was to assess resilience in SCFA production across different lifestyles. SCFAs are a key component of human-microbiome interaction and taxonomic diversity is higher in non-industrial populations. Our pre-specified hypothesis was that resilience would be higher in non-industrial populations. After our first phase of analysis, we uncovered the contradictory results between genus and species level resilience and we hypothesized this was due to reference database bias.

#### Research subjects

All participants from Burkina Faso (n = 90) were healthy volunteers. After filtering the datasets according to our inclusion/exclusion criteria, we analyzed following number of samples from each population: Industrial North American/European—Human Microbiome Project^25^ (Missouri, Texas—USA, n = 50), Oklahoma^3^ (USA, n = 21), Northern Europe^27^ (n = 43); Industrial Central/East Asia—China ^21^ (Guandong Province, China, n = 38), Tokyo ^20^ (Japan, n = 32), Astana ^24^ (Kazakhstan, n = 26); Pastoral—Mongolia^23^ (Khentii Province, n = 50); Rural Agriculturalist—Burkina Faso (n = 90), Madagascar (n = 50); Hunter Gatherer—Matses^3^ (Peru, n = 25), Hadza^22^ (Tanzania, n = 26).

#### Experimental design

Human fecal samples were collected with informed consent from resident volunteers of a single village in central Burkina Faso under the ethics review committee of Centre MURAZ, a national health research institute in Burkina Faso (IRB ID No. 31/2016/CE-CM); the US-based University of Oklahoma (OU) researchers received these samples de-identified, omitting individual names, however, with consent, sex, age, education level, and residential area was recorded for each individual and is reported in Data File S2. OU IRB deemed this project consistent with US policy 45 CFR 46.101(b) exempt category 4 (OU IRB 6976). Gut metagenomic data were generated as given in Borry et al.^37^. All experiments were performed in accordance with relevant guidelines and regulations, with the University of Oklahoma Institutional Biosafety Committee approving the chemical assays (IBC #1044).

#### Randomization

We randomly subsampled 50 individuals, in R, from each of the Madagascar, Human Microbiome Project, and Mongolian datasets, due to the much higher numbers of individuals in these studies as compared to the remaining datasets. We did not want to skew the different lifestyle groups with overrepresentation from a single dataset.

### Statistical analysis

#### Bioinformatic processing

Metagenomic reads for the following datasets were downloaded from either the NCBI Sequence Read Archive or European Nucleotide Archive: hunter-gatherers (Matses from Peru^3^ and Hadza from Tanzania^22^), pastoralists (residents of Khentii region, Mongolia^23^), rural-agriculturalists (Madagascar^26^), industrial European/North American populations (Human Microbiome Project^25^, Europe^21,27^, and Oklahoma, USA^3^), and industrial Central/East Asian populations (Japan^20^, China^21^, and Kazakhstan^24^). Accession numbers can be found in Data File S3.

All metagenomic data (newly generated from Burkina Faso and downloaded) was processed as follows: AdapterRemoval v2^43^ was used to quality filter and merge reads (quality score > 30, maxns = 0, minlength > 50, minalignmentlength = 10). The resulting FASTQ files (forward, reverse, and merged) were used as input for HUMAnN2^28^ with default parameters and using the UniRef50 database^44^. Briefly, pangenomes are generated for each species identified from metagenomic reads using MetaPhlAn2^45^. Metagenomic reads are mapped against those pangenomes to identify genes; reads not mapping to any pangenome are then mapped against the UniRef50 database^44^ to identify ‘unclassified’ genes. Reads not mapping to neither the pangenomes nor UniRef50 database are termed ‘UNAMAPPED’. Gene abundance is normalized to reads per kilobase (RPKs) to account for differences in reference database size. The gene family output at the species level from HUMAnN2 was used to perform downstream analysis. Each sample’s output was normalized to gene abundance RPKs per 1 million DNA kilobases and then merged into a single file with all samples. The RPK gene abundance is further stratified by the abundance of the gene that is mapped to a species. Phylum, Family, and Genus level tables were created using the humann2_infer_taxonomy script from HUMAnN2. Acetate, Butyrate, and Propionate gene family tables were generated by pulling out all lines that were annotated with the gene names listed above from the respective normalized phylum, family, genus, species normalized gene abundance tables.

The following gene names were used to identify SCFAs: acetate kinase, butyrate kinase, butyryl-CoA: acetate CoA transferase, methylmalonyl-CoA decarboxylase, lactoyl-CoA dehydratase, and CoA-dependent propionaldehyde dehydrogenase. Acetate kinase (*ackA*) is the primary end stage enzyme for acetate synthesis^16^. Butyrate kinase (*buk*) and butyryl-CoA:acetate CoA transferase (*but*) can both catalyze butyrate production from butyryl-CoA^18^. Propionate synthesis can follow three different biochemical pathways: succinate, acrylate, and propanediol depending on the initial substrate^19^. Methylmalonyl-CoA decarboxylase (*mmdA*) is a biomarker for the succinate pathway, lactoyl-CoA dehydratase (*lcdA*) for the acrylate, and CoA-dependent propionaldehyde dehydrogenase (*pduP*) for the propanediol pathway^19^. Taxonomic-gene abundance for *but* and *buk* were combined for butyrate and likewise data for *mmdA*, *lcdA*, and *pduP* combined for propionate to facilitate SCFA comparisons.

Total gene copies per 1 million DNA fragments was calculated in R^46^ using the normalized ‘UNMAPPED’ gene abundance generated from HUMAnN2. The stratified gene family tables, after removal of ‘UNMAPPED’ abundance, were used for the remainder of analysis. The proportion of total gene abundance classified to a taxon was determined by summing the abundance of each gene that mapped to taxon and dividing that value by each sample’s total gene abundance. This was repeated at each taxonomic level. The same procedures were applied to the Acetate, Butyrate, and Propionate gene family tables.

### Ecological Metrics

Richness, phylogenetic diversity (PD), Gini-Simpson, and Hill Numbers^30^ values were generated using the vegan package^47^ in R. Richness was determined as the number of taxa that have a gene abundance value > 0 for each SCFA. PD was calculated using the 16S rRNA gene as a proxy. The 16S rRNA gene for each taxon found across the full dataset was extracted from the EzTaxon^48^ reference database and concatenated into a single 16S rRNA gene FASTA file. This sequences were aligned using mafft^49^ with default parameters and a phylogenetic tree was built using FastTree^50^ in QIIMEv1.9^51^. PD was calculated with resulting tree and gene family tables using vegan. Gini-Simpson and Hill Number values were determined using the gene family tables with vegan. P-values were determined using the Kruskal–Wallis H test and the post-hoc Dunn Test. False discovery rate (FDR) was used to account for multiple testing and all p-values can be found in Data File S1. Plots were generated using ggplot2^52^.

## Supplementary Information

Supplementary Information.

## Data Availability

The Burkina Faso gut microbiome metagenome samples produced in this study are available in NCBI under BioProjectID PRJNA690543.
