# Publisher Correction: Analysis of global human gut metagenomes shows that metabolic resilience potential for short-chain fatty acid production is strongly influenced by lifestyle

**DOI:** 10.1038/s41598-021-89719-x

**Published:** 2021-05-06

**Authors:** David K. Jacobson, Tanvi P. Honap, Andrew T. Ozga, Nicolas Meda, Thérèse S. Kagoné, Hélène Carabin, Paul Spicer, Raul Y. Tito, Alexandra J. Obregon-Tito, Luis Marin Reyes, Luzmila Troncoso-Corzo, Emilio Guija-Poma, Krithivasan Sankaranarayanan, Cecil M. Lewis

**Affiliations:** 1 Laboratories of Molecular Anthropology and Microbiome Research, University of Oklahoma, 101 David L. Boren Blvd, Norman, OK 73019 USA; 2 Department of Anthropology, University of Oklahoma, Norman, OK 73019 USA; 3 Halmos College of Natural Sciences and Oceanography, Nova Southeastern University, Fort Lauderdale, FL 33314 USA; 4 Ministry of Health, Ouagadougou, Burkina Faso; 5 Centre MURAZ Research Institute, Bobo‑Dioulasso, Burkina Faso; 6 Department of Biostatistics and Epidemiology, College of Public Health, University of Oklahoma Health Sciences Center, Oklahoma City, OK 73104 USA; 7 Département de Pathologie Et Microbiologie, Faculté de Médecine Vétérinaire, Université de Montréal, Saint‑Hyacinthe, QC J2S 2M2 Canada; 8 Département de Médecine Sociale Et Préventive, École de Santé Publique de L’université de Montréal, Montréal, QC H3N 1X9 Canada; 9 Centre de Recherche en Santé Publique (CReSP) de l’université de Montréal et du CIUSS du Centre Sud de Montréal, Montréal, QC H3N 1X9 Canada; 10 Center for Applied Social Research, University of Oklahoma, Norman, OK 73019 USA; 11 Centro Nacional de Salud Publica, Instituto Nacional de Salud, Lima, Perú; 12 Facultad de Medicina, Universidad Nacional Mayor de San Marcos, Lima, Perú; 13 Centro de Investigación de Bioquímica Y Nutrición, Facultad de Medicina Humana, Universidad de San Martín de Porres, Lima, Perú; 14 Department of Microbiology and Plant Biology, University of Oklahoma, Norman, OK 73019 USA

Correction to: *Scientific Reports* 10.1038/s41598-021-81257-w, published online 18 January 2021

This Article contains an error, where Supplementary Data files 1-3 were omitted. These files appear below.

## Supplementary Information

Supplementary Data 1.

Supplementary Data 2.

Supplementary Data 3.

