# Supplementary material for: Analysis of global human gut metagenomes shows that metabolic resilience potential for short-chain fatty acid production is strongly influenced by lifestyle
Source: Sci Rep. 2021 Jan 18;11:1724. doi: 10.1038/s41598-021-81257-w (PMC7813856; doi:10.1038/s41598-021-81257-w)
Supplement: Supplementary file 1 — Supplementary Information. [file 41598_2021_81257_MOESM1_ESM.docx]

**Supplementary Materials**

Analysis of global human gut metagenomes shows that metabolic resilience potential for short-chain fatty acid production is strongly influenced by lifestyle

David K. Jacobson,^1,2^ Tanvi P. Honap,^1,2^ Andrew T. Ozga,^3^ Nicolas Meda,^4^ Thérèse S. Kagoné,^5^ Hélène Carabin,^6^ Paul Spicer,^2,7^ Raul Y. Tito,^2^ Alexandra J. Obregon-Tito,^2^ Luis Marin Reyes,^8^ Luzmila Troncoso-Corzo,^9^ Emilio Guija-Poma,^10^ Krithi Sankaranarayanan,^1,11^ and Cecil M. Lewis, Jr.^1,2^*

^1^Laboratories of Molecular Anthropology and Microbiome Research, University of Oklahoma, Norman, OK, USA 73019.

^2^Department of Anthropology, University of Oklahoma, Norman, OK, USA 73019.

^3^Halmos College of Natural Sciences and Oceanography, Nova Southeastern University, Fort Lauderdale, FL, USA 33314.

^4^Ministry of Health, Ouagadougou, Burkina Faso.

^5^Centre MURAZ Research Institute, Bobo-Dioulasso, Burkina Faso.

^6^Département de Pathologie et de Microbiologie, Faculté de Médecine vétérinaire-Université de Montréal, Saint-Hyacinthe, Canada, QC J2S 2M2.

^7^Center for Applied Social Research, University of Oklahoma, Norman, OK, USA 73019.

^8^ Centro Nacional de Salud Publica, Instituto Nacional de Salud, Lima, Perú.

^9^ Facultad de Medicina, Universidad Nacional Mayor de San Marcos, Lima, Perú.

^10^Centro de Investigación de Bioquímica y Nutrición, Facultad de Medicina Humana, Universidad de San Martín de Porres, Lima, Perú.

^11^ Department of Microbiology and Plant Biology, University of Oklahoma, Norman, OK 73019.

**Corresponding Author**

Cecil M. Lewis, Jr.

101 David L. Boren Blvd, Norman, OK, 73019

cmlewis@ou.edu


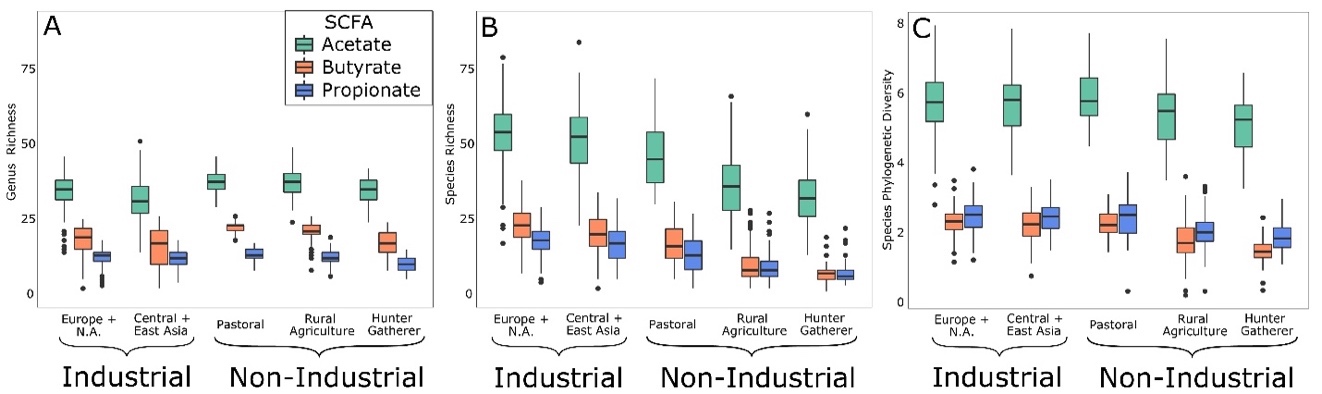


**Fig. S1. Taxonomic Diversity of SCFA-encoding Taxa.** Genus richness (**A**), species richness (**B**), and species phylogenetic diversity (**C**) for bacteria encoding the different SCFAs. **A**) Pastoralists and rural agriculturalists have higher genus richness for acetate and butyrate, hunter-gatherers have significantly lower genus richness for propionate. **B**) Species richness is significantly lower in non-industrial populations for each SCFA. **C)** PD is lower in rural-agriculturalists and hunter gatherers compared to industrial populations. FDR adjusted p-values for all statistical comparisons can be found in data file S1.


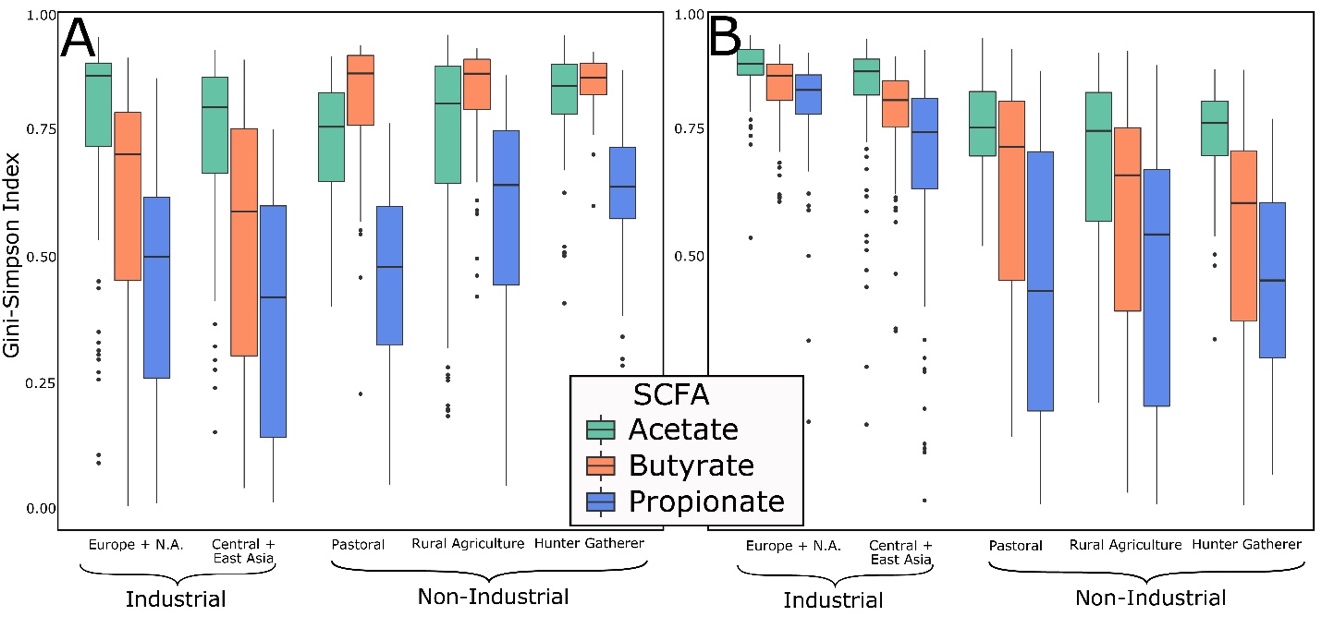


**Fig. S2.** **Gini-Simpson Index Values for Taxa Encoding SCFAs.** Genus (**A**) and species (**B**) level for each SCFA of interest. A) The GS index for butyrate and propionate are higher in the rural agriculturalists and hunter-gatherers compared to the industrial populations at the genus level. B) Each SCFA has significantly lower GS values at the species level in non-industrial populations. FDR adjusted p-values for all statistical comparisons can be found in data file S1.


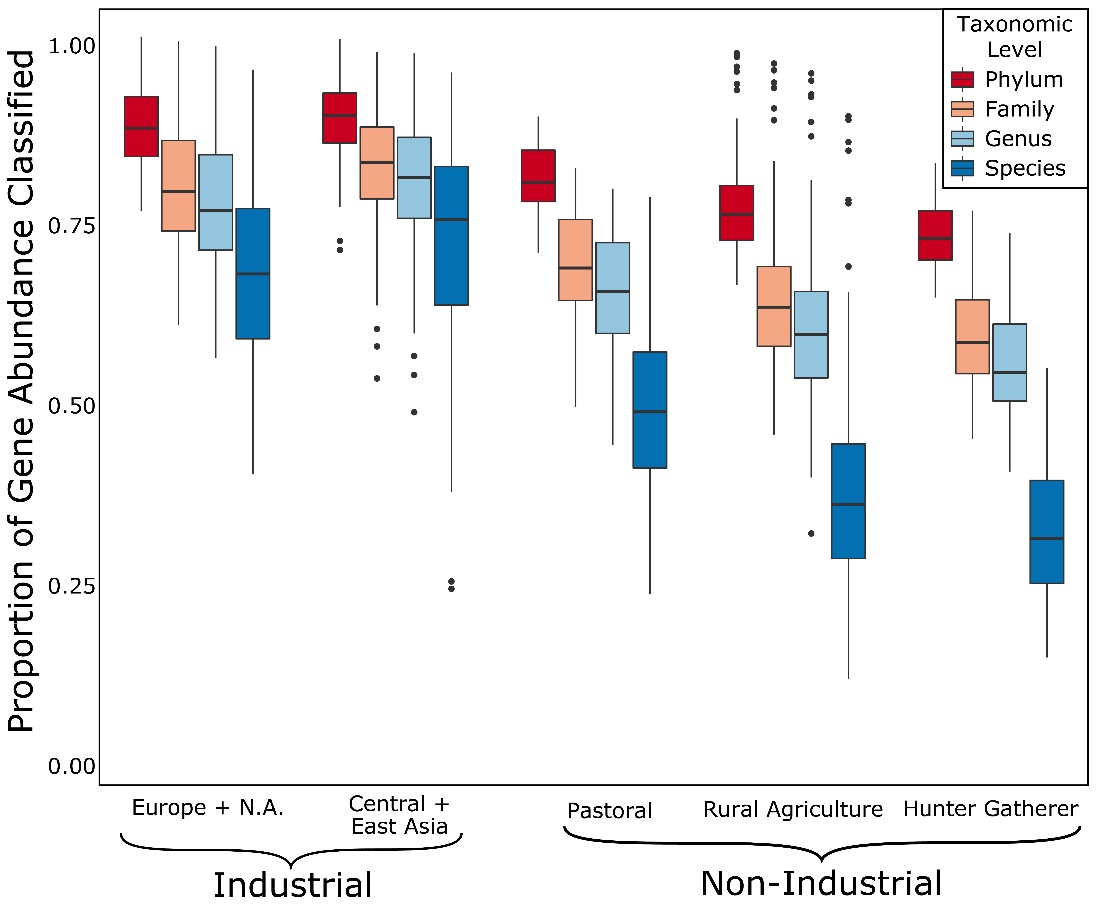


**Fig. S3**. **Proportion of All Genes Classified to a Taxon at Different Phylogenetic Levels**. Classification is significantly worse in the non-industrial populations compared to the industrial populations. FDR adjusted p-values for all statistical comparisons can be found in data file S1.

**
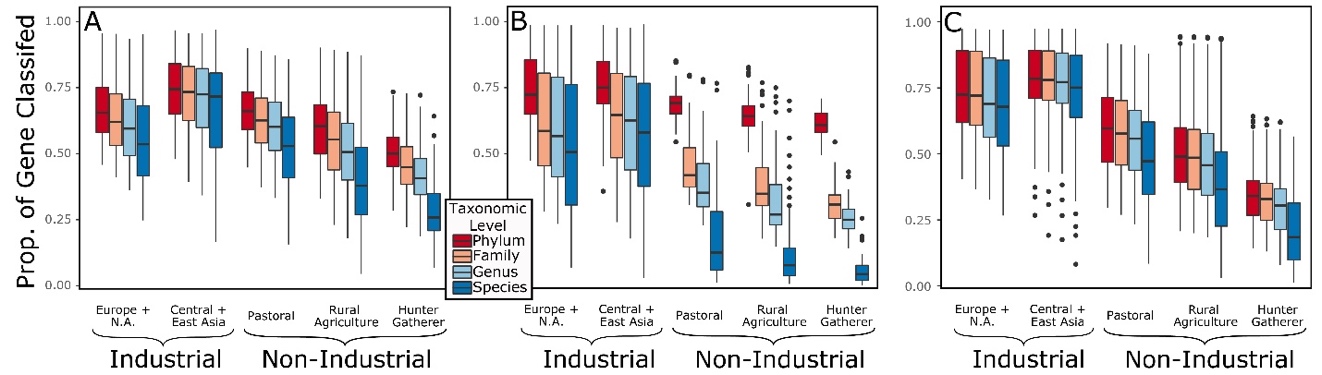
**

**Fig. S4. Proportion of Genes Classified to a Taxon for Each SCFA:** acetate (**A**), butyrate (**B**), and propionate (**C**). Rural agriculturalists and hunter-gatherers have significantly lower proportion of genes mapping to a taxon at each taxonomic level for each SCFA. FDR adjusted p-values for all statistical comparisons can be found in data file S1.

**Table S1. Genera Involved in SCFA Synthesis.** Genera and pathways previously identified in SCFA production.

**Table S2.** **Proportional Contribution to Total SCFA Gene Abundance**. The total SCFA abundance is contributed in the following ratio 0.600:0.215:0.184 for Acetate:Butyrate:Propionate. This follows an expected ratio of 0.6:0.2:0.2 reported in previous studies.

**Table S3.** **Mean and median richness of selected SCFA-producing genera in each lifestyle category.** Number of species found to encode each respective SCFA within different genera of interest. *Bacteroides* and *Clostridium* are at high abundance in industrial gut microbiomes and have many species within each genus, which drives of species richness in industrial populations.

**Data File S1. FDR adjusted p-values for all pairwise comparisons.**

**Data File S2. Novel Burkina Faso Samples.** Samples from Burkina Faso used in our analysis. Gene abundance and proportion of gene abundance mapping to taxa provided

**Data File S3. Non-Burkina Faso samples used in our analysis.** Gene abundance and proportion of gene abundance mapping to taxa provided
